# Growth arrest DNA damage-inducible gene 45 gamma expression as a prognostic and predictive biomarker in hepatocellular carcinoma

**DOI:** 10.18632/oncotarget.4446

**Published:** 2015-06-29

**Authors:** Da-Liang Ou, Song-Kun Shyue, Liang-In Lin, Zi-Rui Feng, Jun-Yang Liou, Hsiang-Hsuan Fan, Bin-Shyun Lee, Chiun Hsu, Ann-Lii Cheng

**Affiliations:** ^1^ Graduate Institute of Oncology, College of Medicine, National Taiwan University, Taipei, Taiwan; ^2^ National Center of Excellence for Clinical Trial and Research, National Taiwan University Hospital, Taipei, Taiwan; ^3^ Institute of Biomedical Sciences, Academia Sinica, Taipei, Taiwan; ^4^ Graduate Institute of Clinical Laboratory Sciences and Medical Biotechnology, College of Medicine, National Taiwan University, Taipei, Taiwan; ^5^ Department of Oncology, National Taiwan University Hospital, Taipei, Taiwan; ^6^ Institute of Cellular and System Medicine, National Health Research Institutes, Zhunan, Taiwan; ^7^ Department of Internal Medicine, National Taiwan University Hospital, Taipei, Taiwan; ^8^ Graduate Institute of Toxicology, College of Medicine, National Taiwan University, Taiwan

**Keywords:** GADD45γ, hepatocellular carcinoma (HCC), sorafenib, CCAAT/enhancer binding protein (C/EBP), survivin

## Abstract

Growth arrest DNA damage-inducible gene 45 (GADD45) family proteins play a crucial role in regulating cellular stress responses and apoptosis. The present study explored the prognostic and predictive role of GADD45γ in hepatocellular carcinoma (HCC) treatment. GADD45γ expression in HCC cells was examined using quantitative reverse transcription-PCR (qRT-PCR) and Western blotting. The control of GADD45γ transcription was examined using a luciferase reporter assay and chromatin immunoprecipitation. The *in vivo* induction of GADD45γ was performed using adenoviral transfer. The expression of GADD45γ in HCC tumor tissues from patients who had undergone curative resection was measured using qRT-PCR. Sorafenib induced expression of GADD45γ mRNA and protein, independent of its RAF kinase inhibitor activity. GADD45γ induction was more prominent in sorafenib-sensitive HCC cells (Huh-7 and HepG2, IC_50_ 6–7 μM) than in sorafenib-resistant HCC cells (Hep3B, Huh-7R, and HepG2R, IC_50_ 12–15 μM). Overexpression of GADD45γ reversed sorafenib resistance *in vitro* and *in vivo*, whereas GADD45γ expression knockdown by using siRNA partially abrogated the proapoptotic effects of sorafenib on sorafenib-sensitive cells. Overexpression of survivin in HCC cells abolished the antitumor enhancement between GADD45γ overexpression and sorafenib treatment, suggesting that survivin is a crucial mediator of antitumor effects of GADD45γ. GADD45γ expression decreased in tumors from patients with HCC who had undergone curative surgery, and low GADD45γ expression was an independent prognostic factor for poor survival, in addition to old age and vascular invasion. The preceding data indicate that GADD45γ suppression is a poor prognostic factor in patients with HCC and may help predict sorafenib efficacy in HCC.

## INTRODUCTION

Sorafenib, a multikinase inhibitor, is the current standard systemic therapy for patients with advanced hepatocellular carcinoma (HCC) [1]. The major mechanism of action includes inhibition of the Raf/mitogen-activated protein kinase-extracellular signal-regulated kinase (MEK)/extracellular signal-regulated kinase (ERK) signaling and inhibition of tumor angiogenesis [2]. However, the antitumor mechanisms of sorafenib may involve complex interactions in cellular signaling pathways, which are independent of the inhibitory effects of sorafenib on Raf/MEK/ERK activities [3–6]. Clarification of these “off-target effects” of sorafenib will facilitate identification of predictive biomarkers for treatment efficacy and combination therapy design for HCC [3].

Growth arrest DNA damage-inducible gene 45 (GADD45) family proteins have been reported to play essential roles in cellular stress response, survival, senescence, and apoptosis regulation [7]. Both tumor-promoting and tumor-suppressing effects of GADD family proteins have been reported, depending on the cell types tested, the types of oncogenic stresses, and the interaction with other cellular signaling pathways [7]. GADD45 family proteins are frequently underexpressed in various types of cancers including HCC [8, 9]. The induction of GADD45 expression in the liver may facilitate the regulation of liver regeneration after hepatectomy or chemically induced liver injury [10, 11]. The role of GADD45 expression in hepatocarcinogenesis remains undetermined.

GADD45 family proteins are also crucial mediators of genotoxic stress-induced apoptosis and transforming growth factor-*β*-induced apoptosis [12, 13]. Induction of GADD45 expression can enhance the therapeutic efficacy of cytotoxic agents in cancer cells [14, 15]. We previously reported that GADD45*β* expression was induced in HCC cells after sorafenib treatment, and this induction was an important predictor of sorafenib sensitivity in HCC cells [16]. We hypothesized that GADD45 family proteins may serve as predictive biomarkers for efficacy of molecular targeted therapy for HCC.

In the present study, we explored the biological significance of GADD45γ expression in HCC tumor tissues, and the potential predictive value of GADD45γ induction in HCC cells on the efficacy of sorafenib treatment. GADD45γ has been reported to be a tumor suppressor in multiple cancer types, and it can induce growth arrest and apoptosis in response to environmental stress [17]. Furthermore, GADD45γ expression was suppressed in HCC, but the clinical significance was unclear [18]. The regulatory mechanisms of GADD45γ expression in response to sorafenib treatment was also explored.

## RESULTS

### Induction of GADD45γ expression in HCC cells by sorafenib correlated with sorafenib efficacy

**Figure 1:**
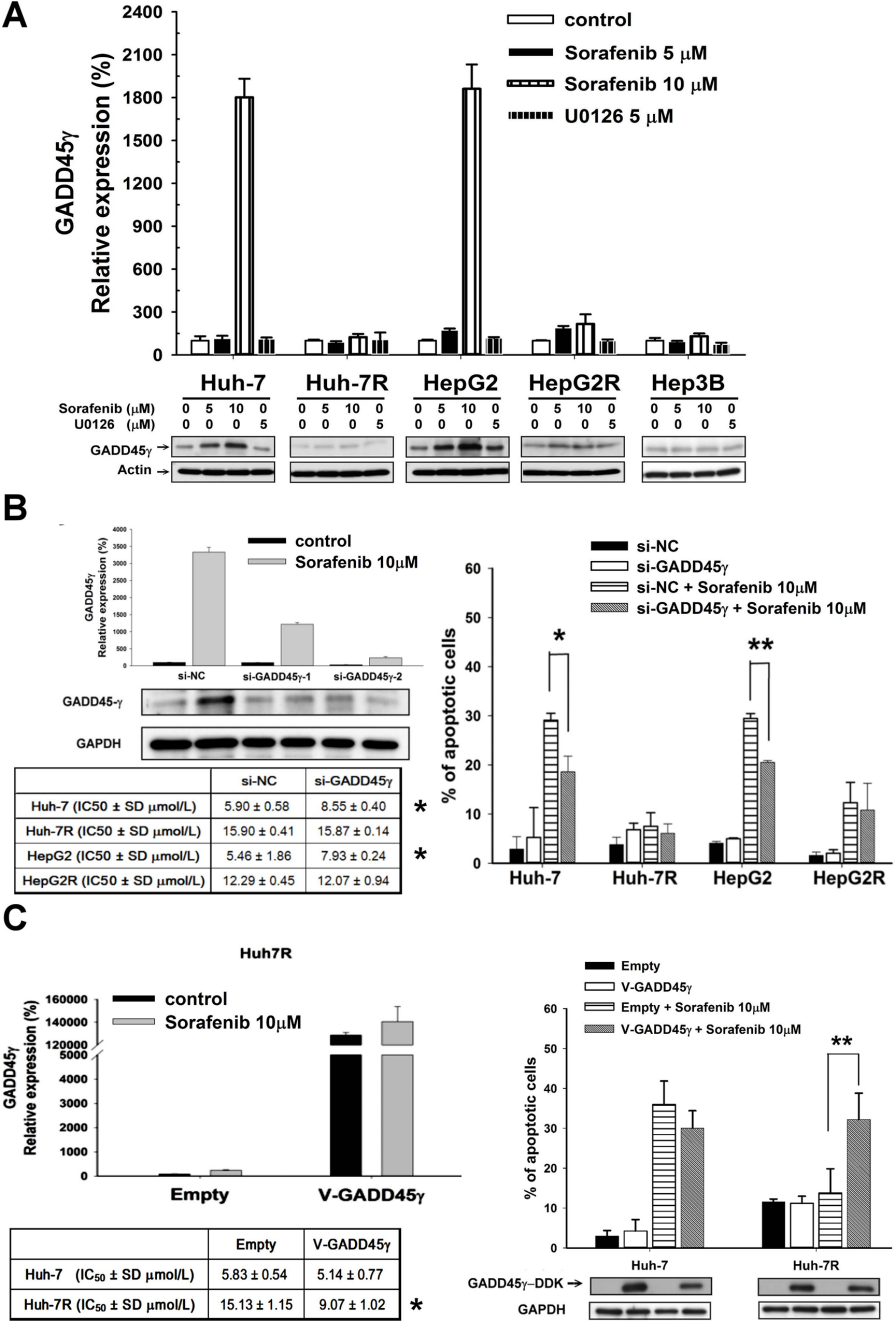
*In vitro* studies on the effects of GADD45γ expression on sorafenib efficacy **A.** GADD45γ mRNA and protein induction after sorafenib, which was independent of cellular RAF/ERK activity, correlated with sorafenib efficacy. HCC cells were treated with sorafenib or U0126 for 24 hours. GADD45γ mRNA and protein levels were assessed using real-time qRT-PCR and Western blotting. Whole cell lysates after drug treatment were examined using Western blotting. **B.** GADD45γ knockdown increased the resistance of sorafenib-sensitive HCC cells to sorafenib. In the left panel, efficacy of GADD45γ knockdown was measured using qRT-PCR and Western blotting. Huh-7 cells were transfected with siRNA directed against GADD45γ (si-GADD45γ-1 and si-GADD45γ-2) or a negative-control (si-NC) siRNA and treated with sorafenib (10 μM) for 48 hours. IC_50_ of sorafenib with or without GADD45γ knockdown were assessed using MTT analysis. *p < 0.05 compared with si-NC. In the right panel, effects of GADD45γ knockdown on sorafenib-induced apoptosis were assessed using Sub-G1 analysis. Each value is the mean ± SD of three independent experiments. *, *p* < 0.05; **, *p* < 0.01, compared with si-NC + sorafenib 10 μM. **C.** GADD45γ over-expression could reverse sorafenib resistance. In the left panel, efficacy of GADD45γ overexpression was measured using quantitative RT-PCR and Western blotting. Huh-7 and Huh-7R cells were transfected with GADD45γ (V-GADD45γ) or empty vectors and treated with sorafenib (10 μM) or a control. IC_50_ of sorafenib with or without GADD45γ overexpression were assessed using MTT analysis. *p < 0.05 compared with empty vectors. Huh-7 and Huh-7R cells were transfected with GADD45γ or empty vectors and treated with sorafenib for 72 hours. In the right panel, proportions of apoptotic cells are indicated by the percentage of cells in the sub-G1 fraction. Columns, mean of three independent experiments; bars, SD. *, *p* < 0.05; **, *p* < 0.01, compared with Empty + Sorafenib 10 μM.

*In vitro* studies on the effects of GADD45γ expression on sorafenib-sensitive (Huh-7 and HepG2, IC_50_ 6–7 μM), acquired sorafenib-resistant (Huh-7R and HepG2R, IC_50_ 12–15 μM), and intrinsic sorafenib-resistant (Hep3B, IC_50_ 12 μM) HCC cells are summarized in Figure 1. The baseline GADD45γ expression did not vary signficantly between sorafenib-sensitive and sorafenib-resistant HCC cells (Figure 1A). However, GADD45γ induction was more prominent in sorafenib-sensitive HCC cells than in sorafenib-resistant HCC cells and was independent of the MEK/ERK signaling in HCC cells because the RAF inhibitor, ZM336372, or the MEK inhibitor, U0126, could not induce GADD45γ expression (Figure 1A and Supplementary Figure S1).

The suppression of GADD45γ expression by using siRNA increased the resistance to sorafenib in sorafenib-sensitive HCC cells; this was evident with the increased IC_50_ and reduced sorafenib-induced apoptosis (Figure 1B and Supplementary Figure S2A). Overexpression of GADD45γ in acquired sorafenib-resistant HCC cells significantly enhanced the suppression of proliferation and sorafenib-induced apoptosis (Figure 1C and Supplementary Figure S2B).

**Figure 2:**
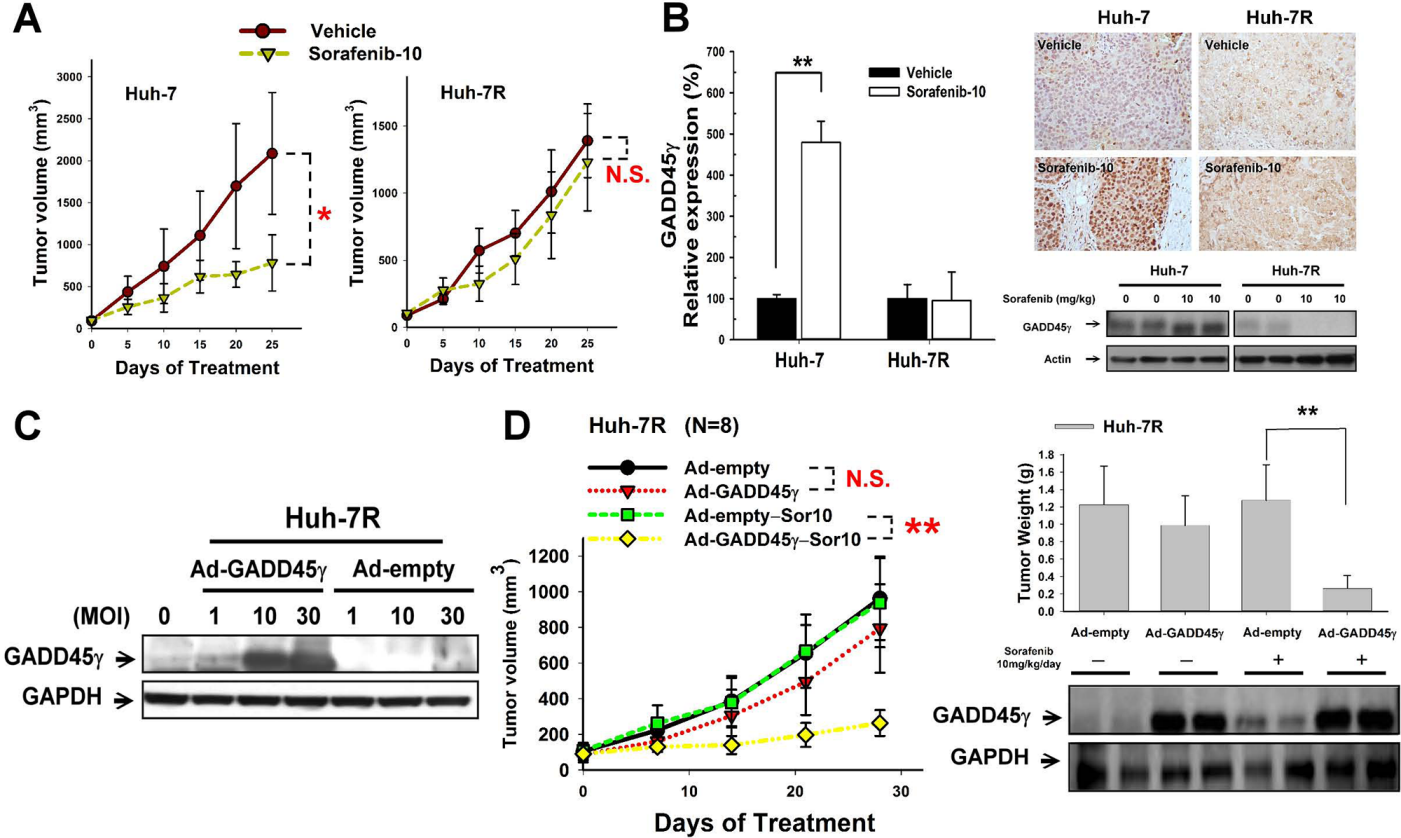
*In vivo* studies on the effects of GADD45γ induction on sorafenib efficacy Huh-7 or Huh-7R cells were injected subcutaneously into male BALB/c athymic nude mice. Mice were treated daily by gavage as indicated (Vehicle or sorafenib 10 mg/kg/day (Sor-10)). **A.** Difference in tumor growth between Huh-7 and Huh-7R xenografts after sorafenib treatment (*n* = 5 in each group). **B.** Difference in GADD45γ mRNA and protein expression measured using real-time qRT-PCR, immunohistochemical staining, and Western blotting. GADD45γ mRNA was expressed relative to endogenous AFP expression. **C.** Efficacy of GADD45γ induction by infection with adenoviral vectors. Huh-7R cells were infected with Ad-GADD45γ and a control virus (Ad-empty) at the indicated multiplicity of infection (MOI). Expression of GADD45γ was measured using Western blotting. **D.** GADD45γ induction reversed sorafenib resistance *in vivo*. Huh-7R cells were infected with GADD45γ-expressing or control (10 MOI) adenoviruses and were implanted subcutaneously into BALB/c athymic (nu^+^/nu^+^) mice 24 hours after adenoviral infection. Mice were treated with sorafenib (10 mg/kg/day) or a vehicle (*n* = 8 in each group). Tumors were harvested after 28 days of sorafenib treatment. Difference in tumor growth and difference in tumor weight and GADD45γ expression at the end of sorafenib treatment are shown. **, *p* < 0.01, compared with the control group.

*In vivo* studies determining the significance of GADD45γ induction in sorafenib-induced apoptosis are summarized in Figure 2. Sorafenib treatment (10 mg/kg/day) inhibited tumor growth in Huh-7 but not in Huh-7R xenografts (Figure 2A). Inhibition of tumor growth in Huh-7 xenografts was associated with the induction of GADD45γ expression, increased tumor cell apoptosis (TUNEL assay), and reduced tumor angiogenesis (microvessel density) (Figure 2B and Supplementary Figure S3A and S3C). GADD45γ overexpression in Huh-7R xenografts, achieved using adenoviral transfer (Figure 2C), did not demonstrate tumor-suppressing effects by itself. However, significant sensitization of the Huh-7R xenograft to sorafenib, increased tumor cell apoptosis (TUNEL assay), and reduced tumor angiogenesis were observed (Figure 2D and Supplementary Figure S3D and S3F). The body weight of animals with or without sorafenib treatment or GADD45γ overexpression did not vary significantly (Supplementary Figure S3B and S3E). This indicated that GADD45γ induction may act in a synthetic-lethal manner to enhance the antitumor activity of sorafenib in HCC cells.

### Survivin is a crucial downstream mediator of GADD45γ induction to reverse sorafenib resistance

**Figure 3:**
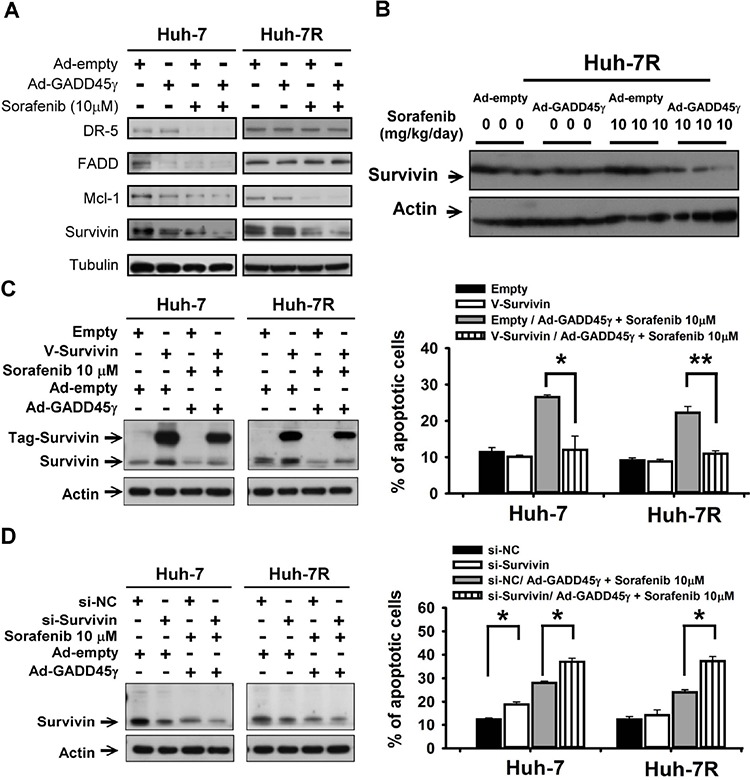
Survivin is a crucial downstream mediator of GADD45γ induction to reverse sorafenib resistance **A.** Effects of GADD45γ induction to reverse sorafenib resistance on apoptosis-related proteins in Huh-7R cells. Huh-7 and Huh-7R cells were infected with GADD45γ-expressing (Ad-GADD45γ) or control (Ad-empty) adenoviruses and sorafenib (10 μM) for 48 hours. Whole cell lysates were subjected to Western blotting. **B.** Expression of survivin in xenograft experiments described in Figure 2D. **C.** Survivin was overexpressed by transfecting pCMV6-Myc-DDK-survivin (V-Survivin) into Huh-7 and Huh-7R cells. The cells were then treated with sorafenib (10 μM) and GADD45γ-expressing (Ad-GADD45γ) or control (Ad-empty) adenoviruses. **D.** Survivin knockdown enhanced the efficacy of GADD45γ induction combined with sorafenib. Huh-7 and Huh-7R cells were transfected with si-survivin or scrambled siRNA (si-NC) for 12 hours. The cells were then treated with sorafenib (10 μM) and GADD45γ-expressing or control adenoviruses. In **C.** and **D.** whole-cell lysates were collected for Western blotting after 48-hour drug treatment. The percentages of apoptotic cells were measured using flow cytometry after 72-hour drug treatment. Columns, mean of three independent experiments; bars, SD. *, *p* < 0.05; **, *p* < 0.01.

To explain the antitumor enhancement between GADD45γ induction and sorafenib, we assessed the molecules that have been linked to sorafenib resistance, including survivin, DR5/FADD (the TRAIL pathway), and Mcl-1, by using Western blotting [19–21]. Survivin was selected as the candidate mediator to explain the apoptosis-enhancing effects of GADD45γ because the variation in survivin expression was compatible with the enhanced antitumor efficacy between sorafenib and GADD45γ induction (Figure 3A and 3B). The baseline survivin expression did not vary signficantly between sorafenib-sensitive and acquired sorafenib-resistant HCC cells (Figure 3A and Supplementary Figure S4A). Survivin overexpression reduced the apoptosis-inducing effects of GADD45γ overexpression combined with sorafenib in the sorafenib-sensitive and sorafenib-resistant HCC cells (Figure 3C, Supplementary Figure S4A–S4C, and S5A). However, suppression of survivin expression by using siRNA knockdown further increased the apoptosis-inducing effects between GADD45γ overexpression and sorafenib (Figure 3D and Supplementary Figure S5B). Mcl-1overexpression, which has been reported as an essential mediator of antitumor effects of sorafenib in HCC, resulted in a similar trend of negating the enhancement between GADD45γ and sorafenib (Supplementary Figure S6). The preceding data supports the role of survivin as an important downstream mediator of antitumor enhancement between GADD45γ overexpression and sorafenib.

### CCAAT/enhancer binding protein as a potential regulator of GADD45γ induction by sorafenib

**Figure 4:**
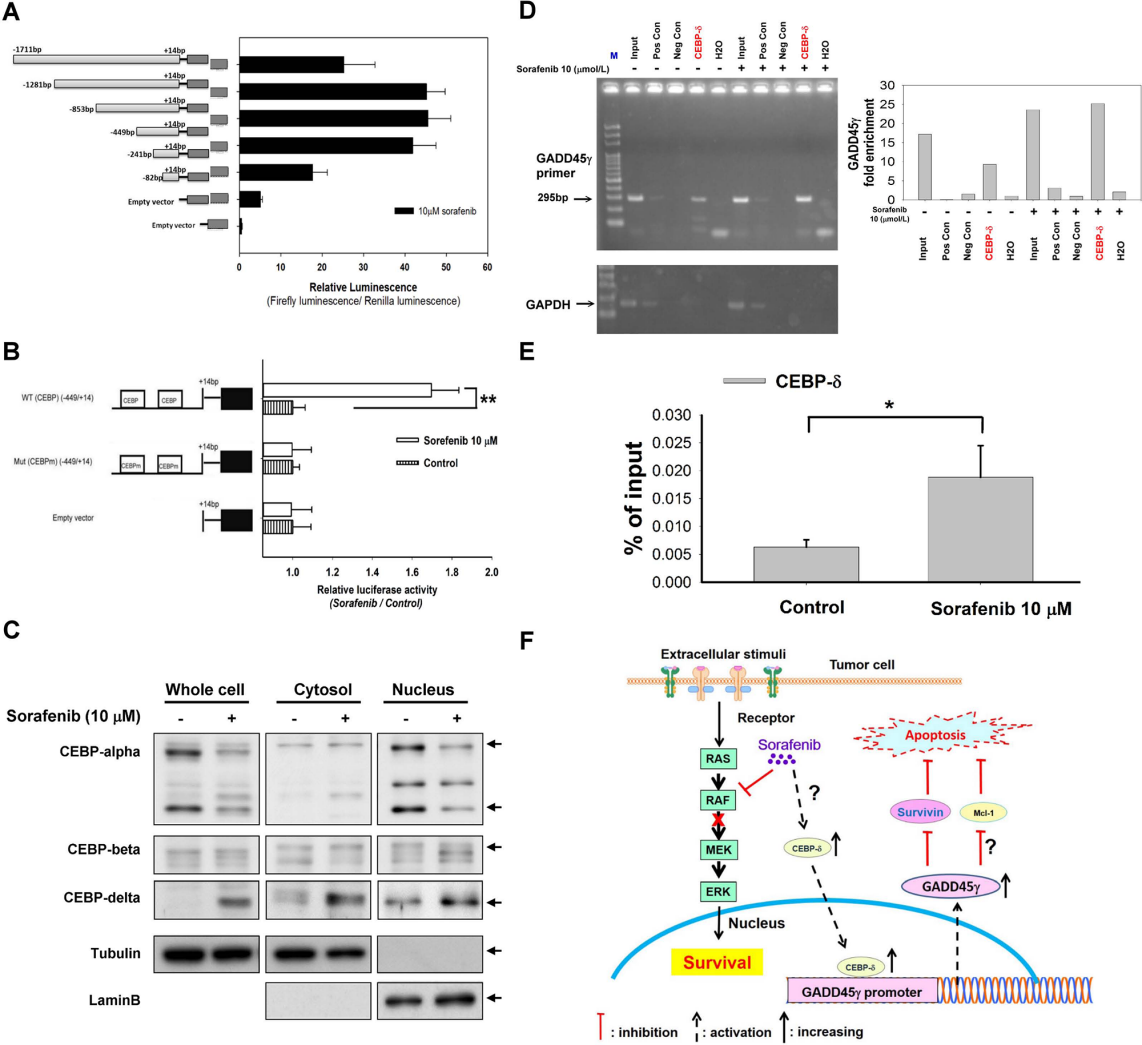
C/EBP as a potential regulator of GADD45γ induction by sorafenib **A.** Localization of the transcriptional regulatory region of the GADD45γ promoter by using 5′-deletion analysis. The 5′-deletion constructs of the GADD45γ promoter (shown on the left) were transfected into Huh-7 cells, and the relative luciferase activity of each promoter fragment after sorafenib treatment is shown on the right. **B.** The GADD45γ promoter constructs with mutations in the C/EBP binding sites (shown on the left) were transfected into Huh-7 cells. The relative luciferase activities of each construct after sorafenib treatment are shown on the right. **, *p* < 0.01, compared with the control group. The results were representative data from three independent experiments. **C.** Analysis of C/EBP isoforms in total cell lysates and nuclear and cytoplasmic fragmentation. Huh-7 cells were treated with sorafenib for 24 hours; lysates were then collected and analyzed by Western blotting. **D.** Chromatin immunoprecipitation assay of the C/EBP-δ association with the GADD45γ promoter-enhancer in Huh-7 cells. Left panel, negative control (Neg Con) was chromatin immunoprecipitated with a normal mouse IgG. Positive control (Pos Con) was chromatin immunoprecipitated with an antiRNA polymerase II antibody. The input was 0.1% of the sonicated chromatin before immunoprecipitation. Right panel, the band intensities were quantified using AutoChemi imaging system (UVP, Cambridge, UK) and signal intensities, representing the DNA content, were normalized to Lane 5 from H_2_O. **E.** The amount of C/EBP-δ binding to the GADD45γ promoter-enhancer was assessed using real-time PCR in chromatin immunoprecipitation assay. *, *p* < 0.05, compared with control. **F.** Proposed mechanisms of the apoptosis-enhancing effects of GADD45γ induction by sorafenib in HCC cells.

We initially analyzed luciferase reporter activities of GADD45γ promoter plasmids (with serial deletions in the 5′-flanking region) because our data indicated that sorafenib induced GADD45γ expression at the transcriptional level. The region of −449/−82 in the 5′-flanking region was crucial for GADD45γ induction by sorafenib (Figure 4A). Two binding sites for the CCAAT/enhancer binding protein (C/EBP) transcriptional factor were identified in this region (−340/−330 and −103/−93) by using the TFSEARCH program (Supplementary Figure S7). Reporters with mutations at these C/EBP binding sites demonstrated no GADD45γ transcriptional activity after sorafenib treatment, further supporting the role of C/EBP in mediating GADD45γ induction by sorafenib (Figure 4B).

Sorafenib treatment increased total and nuclear C/EBP-δ levels (Figure 4C). Increased binding of C/EBP-δ to the GADD45γ promoter was confirmed using chromatin immunoprecipitation (ChIP) (Figure 4D and 4E). The C/EBP-δ RNA levels in HCC cells were independent of sorafenib treatment, suggesting that sorafenib increases C/EBP-δ at the protein level (Supplementary Figure S8). This indicates that C/EBP-δ may help regulate the GADD45γ induction by sorafenib. The potential effects of GADD45γ induction on sorafenib-induced apoptosis in HCC cells was depicted in Figure 4F.

### GADD45γ expression in HCC tumors facilitated the prediction of survival in patients with HCC who had undergone curative surgery

**Figure 5:**
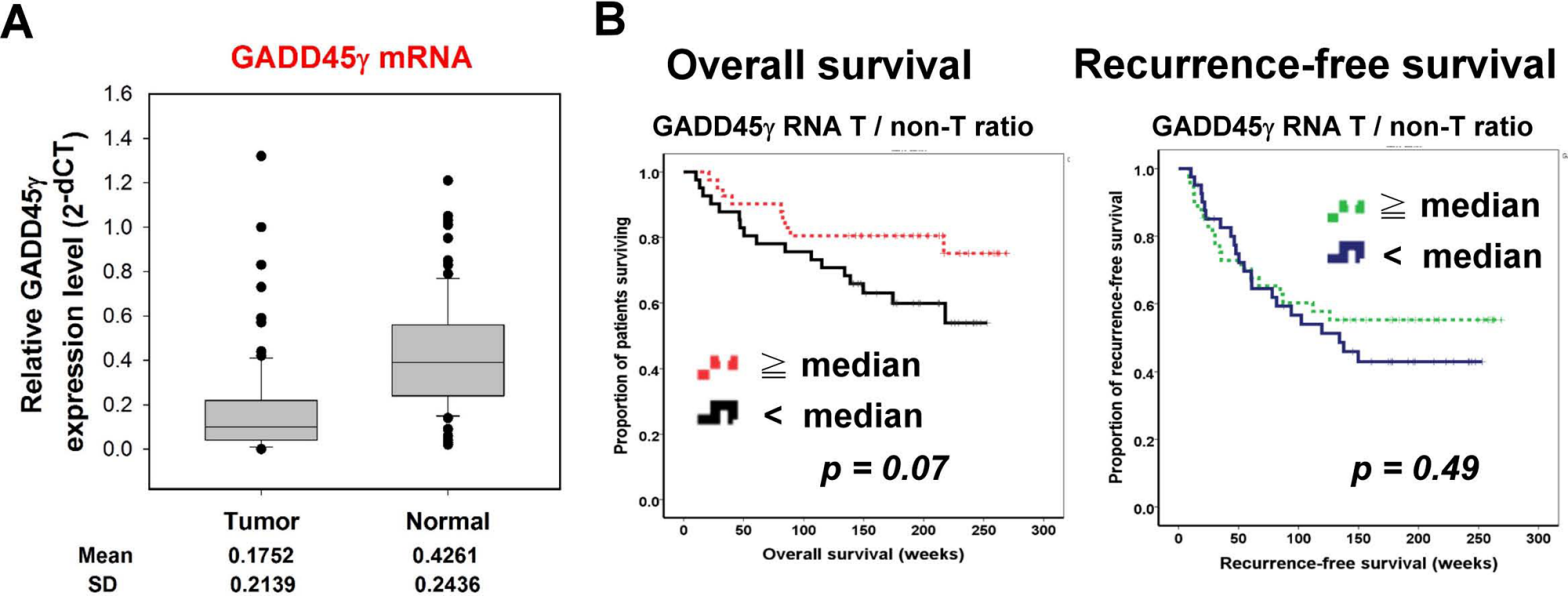
Low GADD45γ expression in HCC tumors predicted poor survival in patients with HCC who had undergone curative surgery **A.** GADD45γ expression was more suppressed in the HCC tumor tissue than in the adjacent normal liver tissue. The expression levels were represented by the relative amount of the target gene (GADD45γ) vs. control gene (HPRT). 2 ^− ΔCT^, where ΔCT = CT (target gene) − CT (control gene). **B.** Kaplan–Meier survival curves of overall survival and recurrence-free survival for patients with HCC whose tumors expressed high vs. low levels of GADD45γ. *, log-rank test (univariate analysis).

GADD45γ mRNA was more suppressed in HCC tumor tissue than in the adjacent nontumor liver tissue, and the median T to non-T ratio of GADD45γ mRNA was 33.8% (0.7%–974.0%) (Figure 5A). The expression levels of GADD45γ did not correlate with any clinicopathological factors, including age, sex, underlying chronic viral hepatitis, presence of cirrhosis, tumor grade and stage, serum α-fetoprotein levels, and the presence of vascular invasion (Table 1). Univariate analysis revealed that low GADD45γ expression was associated with short overall survival (Figure 5B and Supplementary Table S2). After controlling the potential confounding factors by using multivariate analysis, low GADD45γ expression became an independent predictor of poor overall survival, in addition to age and the presence of vascular invasion (Table 1). The hazard ratio of death was 2.94 (95% CI 1.27–6.77, *p* = 0.012) for patients with low GADD45γ expression (tumor to nontumor ratio of < 33.8%). The potential prognostic value of GADD45γ expression was supported by data from the cBio Cancer Genomics Portal (http://cbioportal.org) [22, 23], which revealed a consistent trend of superior survival in patients with GADD45γ overexpression (Supplementary Table S3).

**Table 1:** GADD45γ expression and survival of patients with HCC who had undergone curative surgery

GADD45γ expression and clinical characteristics*			
	GADD45γ RNA T/non-T ratio		*P* value
	< median (*n* = 41)	≧ median (*n* = 41)	
Age (years) (median/ range)	59.0/37–85	65.0/38–86	0.17
Male/ female	29/12	28/13	0.81
HBV/ HCV	24/17	17/24	0.12
Cirrhosis yes/no	19/22	16/25	0.50
AFP (ng/mL) (median/ range)	97.4/2.6 - 173900	22.3/2.2 - 181200	0.64
Stage I/III/IIIA	17/18/6	21/12/8	0.38
MVI yes/no	18/23	17/24	0.82
Tumor grade 1/2/3	0/27/14	3/29/9	0.12
Multi-variate analysis of overall survival and recurrence-free survival**			
	Coefficient	HR (95% C.I.)	*P* value
Overall survival			
MVI (−) vs. (+)	−1.11	0.33 (0.15–0.74)	0.007
Age < 65 y vs. ≥ 65 y	−1.37	0.25 (0.11–0.59)	0.001
GADD45γ RNA T/non-T ratio ≥ median vs. < median	−1.08	0.34 (0.15–0.78)	0.012
Recurrence-free survival			
MVI (−) vs. (+)	−1.01	0.37 (0.16–0.58)	0.004
Age < 65 y vs. ≥ 65 y	−0.89	0.41 (0.25–0.88)	0.007

* Continuous variables were compared using one-way ANOVA; categorical variables were compared using the chi-square and Fisher's exact tests.

** The variables analyzed in the regression model included age, sex, tumor stage (stage IIIA vs. stage I/II), tumor grade (grade 2 vs. 0–1), virus infection (hepatitis B vs. C), α-fetoprotein levels, presence of cirrhosis, and the presence of macrovascular invasion (MVI).

## DISCUSSION

In this study, we demonstrated that GADD45γ expression in HCC cells was associated with the sorafenib sensitivity of HCC cells. The induction of GADD45γ expression can reverse the resistance of HCC cells to sorafenib. In addition, suppression of GADD45γ expression in HCC tumor tissue was observed to be an independent predictor of poor overall survival in patients with HCC who had undergone curative surgery. The results indicate that GADD45γ may act as a prognostic factor and as a marker for response to sorafenib in HCC.

Regulation of GADD45 expression, which involves complex interaction between cellular growth control and stress response, has been extensively studied [24, 25]. Although suppression of GADD45 expression in cancer cells is commonly noted, the clinical significance is unclear. In this study we demonstrated that GADD45γ expression was not associated with other clinicopathological features of HCC, and the suppression of GADD45γ expression was correlated with poor overall survival in patients with HCC who had undergone curative surgery. Our data suggest that GADD45 family proteins may serve as tumor suppressors in carcinogenesis, which is different from the survival-promoting effects of GADD45 in liver regeneration [26, 27]. Because GADD45 family proteins may be regulated by similar cellular mechanisms, it is of value to explore the potential interaction between GADD45γ and other GADD45 family proteins in HCC progression and their prognostic values [28].

Validation of the predictive value of GADD45γ induction in response to sorafenib treatment is challenging. In our studies, the difference in GADD45γ induction after sorafenib treatment varied significantly between sorafenib-sensitive and sorafenib-resistant HCC cells. Therefore, comparison of GADD45γ between pre- and posttreatment tumor samples is essential to validate the concept. Tumor specimens for biomarker analysis were difficult to obtain because of the prevalent clinical diagnosis of HCC [1]. An additional difficulty was obtaining pre- and posttreatment biopsy specimens from patients with advanced HCC because the majority of patients have a high bleeding risk due to the underlying cirrhosis. Development of novel and noninvasive technology may help detect the molecular changes after drug therapy and facilitate the validation of predictive biomarkers for HCC [29, 30].

A major finding in this study is that while GADD45γ overexpression alone had no evident effects on growth or apoptosis induction in HCC cells, GADD45γ overexpression can significantly reverse the resistance of HCC cells to sorafenib *in vitro* and *in vivo*. This finding is consistent with previous studies, which suggest that GADD45γ can enhance the efficacy of cytotoxic therapy and provide opportunities for synthetic lethality-based development of new therapeutic targets [31]. The frequently reported downstream mediators of the proapoptotic effects of GADD family proteins include stress-related mitogen-activated protein kinases (p38 and MKK4) and the proapoptotic proteins Bim and PUMA [32, 33]. The possibility of using GADD45 expression as a marker for drug screening should be explored. A more comprehensive exploration of the signaling network pertinent to the proapoptotic effects of GADD45γ in HCC will facilitate identifying new druggable targets for HCC treatment.

Our results indicate that survivin and Mcl-1 are possible downstream mediators of the antitumor enhancement between GADD45γ and sorafenib. Survivin, which facilitates the integration of the cellular signals determining cellular proliferation, survival, and drug resistance, has been reported as a key regulator of the resistance of HCC cells to various molecular targeted agents [21, 34–36]. GADD45γ may act as a transcriptional coactivator or interact with other cellular signaling pathways, including cell cycle control and stress response, to regulate cell survival. Therefore, it may inhibit survivin expression through both transcriptional and posttranslational mechanisms [37, 38]. On the other hand, survivin expression is regulated by a complex intracellular signaling network and sorafenib may inhibit survivin expression through the downregulation of a mammalian target of rapamycin activity, independent of GADD45γ [39]. Mcl-1 is also a crucial antiapoptotic protein for cell survival maintenance and drug resistance regulation [40–42]. Therapeutic strategies targeting survivin or Mcl-1 have been developed, and additional studies based on these strategies alone or in combination with sorafenib in patients with HCC are warranted [43, 44].

In this study, we demonstrated that the transcriptional factor C/EBP-δ may increase the induction of GADD45γ by sorafenib. Previous studies have indicated that C/EBP-δ has contrasting effects at different stages of carcinogenesis [45]. However, C/EBP-δ can suppress tumor growth by inducing apoptosis, augmenting DNA damage response, and inhibiting cell cycle progression [46, 47]. Furthermore, C/EBP-δ can induce lymphangiogenesis and tumor metastases in response to hypoxia [48, 49]. GADD45γ can also be induced by other transcriptional factors and by the methylation status of the promoter region [50]. Cumulative evidence indicates that GADD45 family proteins may facilitate the integration of cellular response and environmental stress by interacting with different signaling pathways [7]. An improved understanding of the mechanisms regulating GADD45 family protein expression in HCC cells will facilitate clarification on whether GADD45 family proteins are suitable targets for new drug development for HCC.

In conclusion, our study indicates that GADD45γ suppression is a poor prognostic factor in patients with HCC. The induction of GADD45γ expression contributes to sorafenib-induced apoptosis in HCC cells and may serve as a biomarker for the development of new targeted therapy for HCC.

## MATERIALS AND METHODS

### Cell culture

The HCC cell lines, HepG2 and Hep3B, were obtained from the American Type Culture Collection, and the Huh-7 cell line was from the Health Science Research Resources Bank. In this study Huh-7 and HepG2 cells (sorafenib IC_50_ 6–7 μM) were classified as sorafenib-sensitive because previous pharmacokinetic studies have determined the maximal plasma concentration of sorafenib in patients treated by the recommended dosage by Food Drug Administration (400 mg twice daily), was between 5 and 10 μM [51, 52]. Cell lines with acquired resistance to sorafenib, Huh-7R and HepG2R, were generated through continuous sorafenib treatment (up to 10 μM). Cells were cultured in Dulbecco's modified Eagle's medium containing 10% fetal bovine serum, penicillin (100 units/mL), streptomycin (100 μg/mL), L-glutamine (2 mmol/L), and sodium pyruvate (1 mmol/L) at 37°C in a humidified incubator containing 5% CO_2_.

### Quantitative reverse transcriptase polymerase chain reaction

RNA extraction, cDNA synthesis, and cDNA quantification were performed as described previously [16]. The primers for the GADD45γand CEBPδ genes were purchased from Applied Biosystems (ABI TaqMan assay ID: Hs00198672_m1 and Hs00270931_s1). The primers for the hypoxanthine phosphoribosyltransferase gene were used as endogenous controls (see Supplementary Table S1 for the primer sequences). The conditions for PCR were as follows: 50°C for 2 min, 95°C for 10 min, 40 cycles of 95°C for 15 s (denaturation), and 60°C for 1 min (annealing and extension). The relative mRNA amount of the target and control genes was calculated using the ΔCt (threshold cycle) method. Relative expression = 2 ^−ΔCt^, where ΔCt = Ct (target gene) − Ct (control gene).

### Small interfering RNA knockdown

The GADD45γ, Mcl-1, and scrambled nonspecific (negative control) siRNAs were purchased from Ambion (Austin, TX, USA) (see Supplementary Table S1 for the sequences). The si-survivin (catalog number L-003459-00-0005) and scrambled nonspecific (negative control) siRNA (catalog number D-001810-10-20) were purchased from Thermo Scientific (Dharmacon Division) as previously described [34]. siRNA were transfected using the siPORT NeoFx siRNA transfection reagent (Ambion). The transfected HCC cells were subsequently treated with sorafenib (10 μM) for 48 hours and collected for subsequent Western blot or quantitative reverse transcriptase polymerase chain reaction (qRT-PCR) analysis.

### Chromatin immunoprecipitation assay

Huh-7 cells (approximately 5 × 10^6^) with or without sorafenib treatment were used for ChIP, employing an EZ-ChIP assay kit (Millipore, Billerica, MA, USA). The PCR amplification was performed using primers spanning the CEBP sites on the GADD45γ promoter from nucleotides −443 to −61 (see Supplementary Table S1 for sequences), as described previously [16]. The band intensities were quantified using AutoChemi imaging system (UVP, Cambridge, UK), and signal intensities representing DNA content were normalized to H_2_O. The conditions for quantitative PCR were as follows: 50°C for 2 min, 95°C for 10 min, 50 cycles of 95°C for 20 s, and 60°C for 1 min. The relative DNA amount of the target and 0.1% input GADD45γ was calculated using the ΔCt (threshold cycle) method as previously described.

### Tumor xenograft experiments

The protocol for the xenograft experiments was approved by the Institutional Animal Care and Use Committee of the College of Medicine, National Taiwan University. The animal studies were performed according to the criteria outlined in the Guide for the Care and Use of Laboratory Animals prepared by the National Academy of Sciences and published by the National Institutes of Health. Male BALB/c athymic (nu^+^/nu^+^) mice were inoculated subcutaneously with Huh-7 cells or Huh-7R cells (approximately 1 × 10^6^). Huh-7R cells infected with the Ad-GADD45γ or Ad-empty virus were used to determine the effects of GADD45γ overexpression on treatment efficacy. When the tumor volume reached approximately 100 mm^3^ (volume [mm^3^] = [width]^2^ × length × 0.5), the mice were randomized to receive sorafenib (10 mg/kg/day) or vehicle treatment (*n* ≥ 5 in each group). Drug treatments were given daily by gavage. Tumor volume and body weight were recorded every 5 days. Tumor samples, freshly frozen after drug treatment, were collected to measure the levels of pertinent mRNA and proteins by using real-time PCR and Western blot, respectively. The total RNA was extracted from cells of tumor specimens, which was collected using laser capture microdissection [15]. Formalin-fixed and paraffin-embedded tumor samples after drug treatment were collected for immunohistochemical analysis of pertinent protein expression and tumor angiogenesis and TUNEL assay to measure tumor cell apoptosis, as described previously [16].

### Tumor specimens from patients with HCC

Total RNA was extracted from HCC tumors and adjacent liver tissues, which were obtained from the tissue bank of Taiwan Liver Cancer Network (TLCN). Eighty-two patients with American Joint Committee on Cancer (AJCC) stage I to IIIA disease who had undergone curative surgery were included in this study. An informed consent for tissue collection was obtained from each patient prior to surgery. Data of clinicopathological features at surgery and postoperational follow-up were provided by TLCN. Recurrence-free survival time was calculated from the date of surgery to the date of documented HCC tumor recurrence, and overall survival time was calculated from the date of surgery to the date of death or last follow-up. The primers for the GADD45γ gene were purchased from Applied Biosystems (ABI TaqMan assay Hs00198672_m1). Quantification of GADD45γ expression was using the ΔCt (threshold cycle) method described previously and expressed as a tumor ΔCt/nontumor ΔCt (T/non-T) ratio.

### Statistical analysis

For *in vitro* and xenograft experiments, data were representative of three independent experiments. Quantitative data were expressed as mean ± standard deviation (SD). Comparisons were analyzed using the Student *t* test and one-way analysis of variance (ANOVA). Survival time for the human HCC tissue studies was calculated using the Kaplan–Meier method and compared in different subgroups of patients by using the log-rank test. The prognostic significance of individual clinicopathological factors and GADD45γ expression was analyzed using the Cox proportional hazards model. Significance was defined as *p* < 0.05.

## SUPPLEMENTARY MATERIALS AND METHODS FIGURES AND TABLES


