# Supplementary material for: Growth arrest DNA damage-inducible gene 45 gamma expression as a prognostic and predictive biomarker in hepatocellular carcinoma
Source: Oncotarget. 2015 Jun 29;6(29):27953–65. doi: 10.18632/oncotarget.4446 (PMC4695037; doi:10.18632/oncotarget.4446)
Supplement: Supplementary file 1 [file oncotarget-06-27953-s001.pdf]

SUPPLEMENTARY MATERIALS AND METHODS

Chemicals and other reagents

Compounds used in this study included sorafenib (Bayer-Schering Pharma, West Haven, CT), U0126 (MEK kinase inhibitor, Calbiochem, La Jolla, CA), and ZM336372 (Raf kinase inhibitor, Merck KGaA, Darmstadt, Germany). For *in vitro* experiments, the compounds were dissolved in DMSO, and the final concentration of DMSO was kept below 0.1%. For in vivo experiments, sorafenib was dissolved in Cremophor EL/95% ethanol (50:50; Sigma-Aldrich, St. Louis, MO). The antibodies used for Western blotting, immunohistochemical staining, and chromatin immunoprecipitation (ChIP) assays included GADD45γ (Sigma-Aldrich, MO), ERK 2, phospho-ERK 1/2, GAPDH, survivin, Mcl-1, actin (Santa Cruz Biotechnology, Santa Cruz, CA), CEBP-α, CEBP-β (Cell Signaling Technology, Danvers, MA), lamin B, α-tubulin (Calbiochem, La Jolla, CA), CEBP-δ, CD31, GADD45γ (Abcam, Cambridge, MA).

Reporter constructs and assay of the GADD45γ promoter activity

Proximal promoter fragments of GADD45γ, spanning -1711 to +14, were cloned upstream of the luciferase gene in the pGL4.17-base luciferase expression plasmid (Promega). Six different GADD45γ promoter deletion fragments were generated by PCR using the sense and antisense primers listed in supplement. An EcoRV site was incorporated into the sense primers, and a HindIII site was incorporated into the antisense primers. The TFSEARCH program (version 1.3; http://www.cbrc.jp.reserach/db/TFSEARCH.html) was used to identify possible binding sites for transcription factors in the GADD45γ promoter. Gene synthesis was performed to obtain sequences that mutated at CEBP (CEBPm) binding sites (sequences listed in supplementary table 1) from Genomics BioScience and Technology Co., Ltd. (Taiwan). Huh-7 genomic DNA was used as the PCR template. PCR products were digested, purified, and cloned into the corresponding sites of pGL4.17 vector (Promega, Madison, WI). Huh-7 cells were transfected with individual GADD45γ reporter constructs using Lipofectamine 2000 (Invitrogen, Carlsbad, CA) and co-transfected with pGL4.73 [hRluc/SV40], which constitutively expresses renilla luciferase, to normalize transfection efficiency. The promoter activities with or without sorafenib treatment were determined by dual-luciferase assay kit (Promega), according to the manufacturer's directions. The activity levels were expressed relative to a vector control.

Cell viability and apoptosis assays

Cell viability was assessed using an MTT (3-(4, 5-dimethylthiazol-2-yl)-2, 5- diphenyltetrazolium bromide) assay and the IC_{50} values of individual drugs were calculated using CompuSyn software (ComboSyn, Paramus, NJ) based on the changes of absorbance measured by spectrophometry (DTX 880; Beckman Coulter, Fullerton, CA) as previously described [1]. The fraction of apoptotic cells after drug treatment was assessed by sub-G1 fraction analysis and Annexin V staining using flow cytometry as previously described [1].

Annexin V assay

The fraction of apoptotic cells after drug treatment was assessed by Annexin V analysis using flow cytometry. HCC cells were treated with the drugs for 48 h at indicated concentrations, and the cells were trypsinized and collected. For Annexin V analysis, the cells were centrifuged at 300 × g for 5 min at room temperature, and the cell suspension was stained with Annexin V-FITC (Annexin V assay kit, BD Biosciences Pharmingen) and propidium iodide at room temperature for at least 15 min in the dark. The cells were then analyzed by FACScan flow cytometer and Cell Quest program. The proportion of apoptotic cells was the proportion of cells stained with Annexin V. Columns, mean of three independent experiments; bars, SD. *, $p < 0.05$; **, $p < 0.01$.

Western blot analysis

Whole cell lysates of HCC cells after drug treatment were prepared and quantified. Nuclear and cytoplasmic fractions were extracted with a CMN Compartment Protein Extraction kit (BioChain, Hayward, CA). SDS-PAGE and Western blot analysis were performed to measure protein expression. Signals were visualized using a UVP Imaging System (UVP, Upland, CA) or with X-ray film.

Overexpression of GADD45γ and survivin *in vitro* and *in vivo*

For *in vitro* studies, HCC cells were transiently transfected with the pCMV6- GADD45γ-Myc-DDK vector (RC201364; Origene Technologies, Rockville, MD), pCMV6-survivin-Myc-DDK vector (RC205935; Origene Technologies), pCMV6-Mcl-1-Myc-DDK vector (RC200521; Origene Technologies) or empty vector (pCMV6 vector; Origene Technologies, Rockville, MD). Twenty-four hours after transfection, cells were

treated with sorafenib (10 μM) or control. Effects of GADD45γ,survivin, or Mcl-1 overexpression on the sensitivity of HCC cells to sorafenib were measured by flow cytometry. For *in vivo* studies, the human GADD45γ cDNA was sub-cloned into the adenovirus shuttle vector. A recombinant adenovirus (Ad-GADD45γ) was generated by homologous recombination and amplified in human embryonic kidney 293 cells. The adenovirus with no insert (Ad-empty) was used as a control. Viruses were purified by CsCl density gradient centrifugation, and viral titer was determined as previously described [2, 3]. Purified virus was stored in 10 mmol/l Tris–HCl (pH 8.0), 2 mmol/l MgCl2, and 4% (vol/vol) sucrose at –80°C until used for the experiments. For infection, Huh-7R cells (~3 × 10^6) were infected 24 hours before inoculation into mice with the Ad-GADD45γ or Ad-empty virus at the 10 multiplicity of infection (10 MOI).

REFERENCES

1. Ou DL, Shen YC, Liang JD, Liou JY, Yu SL, Fan HH, et al. Induction of Bim expression contributes to the anti-tumor synergy between sorafenib and mitogen-activated protein kinase/extracellular signal-regulated kinase kinase inhibitor CI-1040 in hepatocellular carcinoma. Clin Cancer Res. 2009; 15:5820–5828.
2. Chang TC, Huang CJ, Tam K, Chen SF, Tan KT, Tsai MS, et al. Stabilization of hypoxia-inducible factor-1{alpha} by prostacyclin under prolonged hypoxia via reducing reactive oxygen species level in endothelial cells. J Biol Chem. 2005; 280:36567–36574.
3. Shyue SK, Tsai MJ, Liou JY, Willerson JT, Wu KK. Selective augmentation of prostacyclin production by combined prostacyclin synthase and cyclooxygenase-1 gene transfer. Circulation. 2001; 103:2090–2095.

SUPPLEMENTARY FIGURES AND TABLES

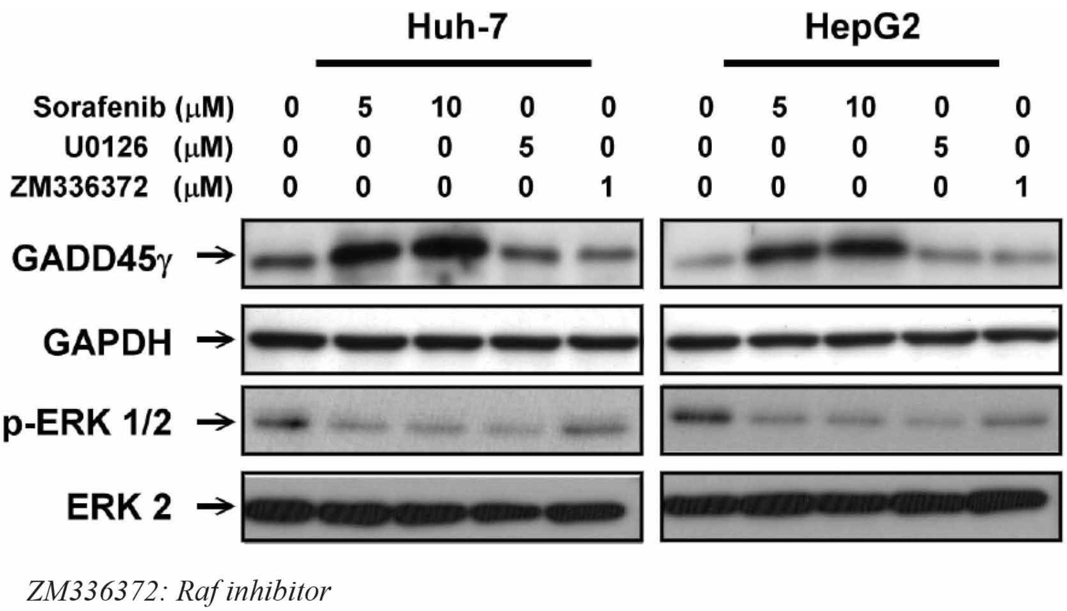

Supplementary Figure S1: Effects of sorafenib and the Raf kinase inhibitor ZM336372 on GADD45γ induction in sorafenib-sensitive Huh-7 and HepG2 cells. Cells were treated with drugs (as indicated), and levels of p-ERK 1/2, ERK 2 and GADD45γ proteins were measured by Western blotting.

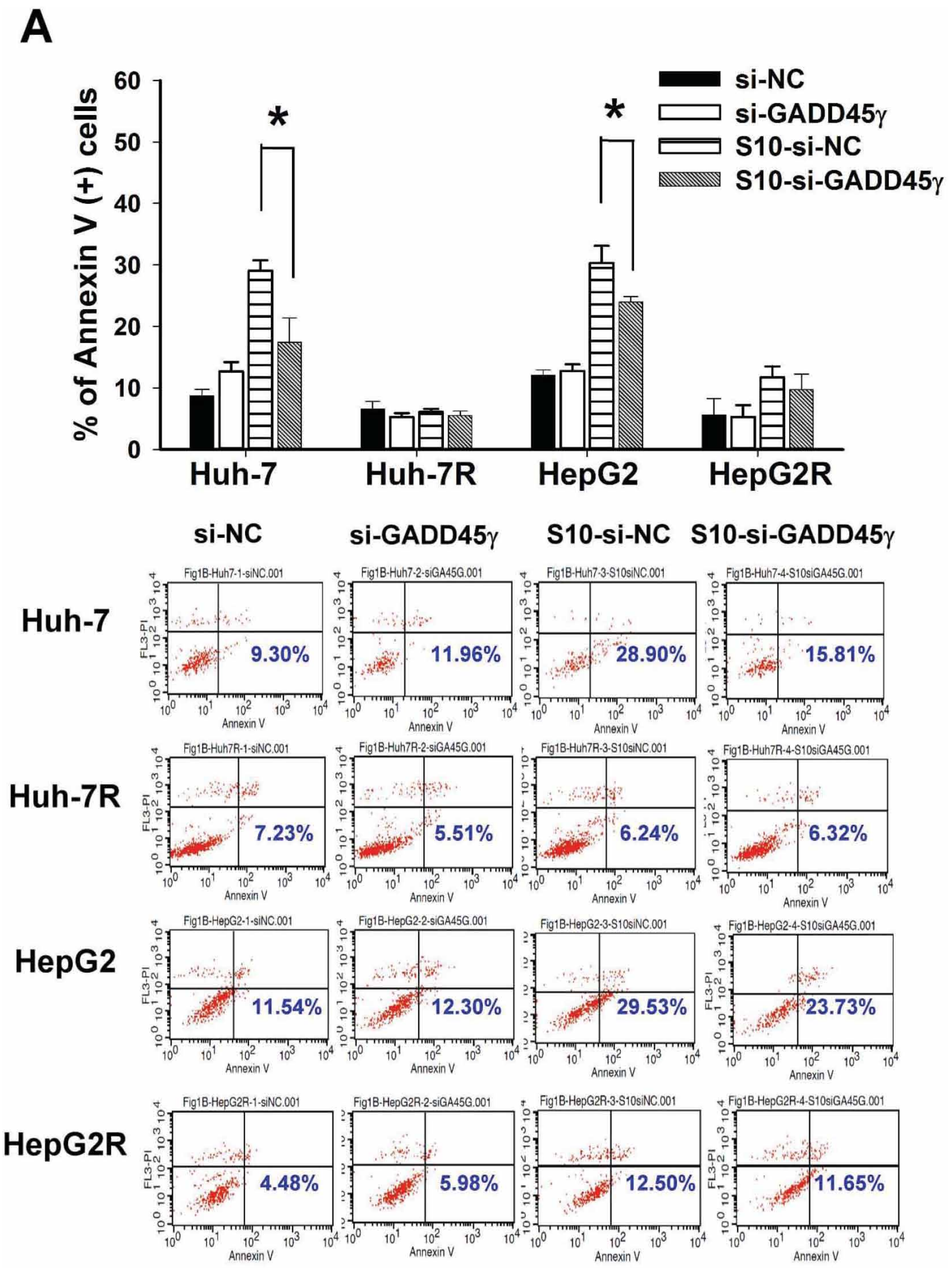

Supplementary Figure S2: Effects of GADD45γ expression on sorafenib efficacy by Annexin V/PI double staining. **A.** GADD45γ knockdown increased the resistance of sorafenib-sensitive HCC cells to sorafenib. HCC cells were transfected with siRNA directed against GADD45γ or a negative-control (NC) siRNA and treated with sorafenib (10 μM; S10) for 48 hours. (*Continued*)

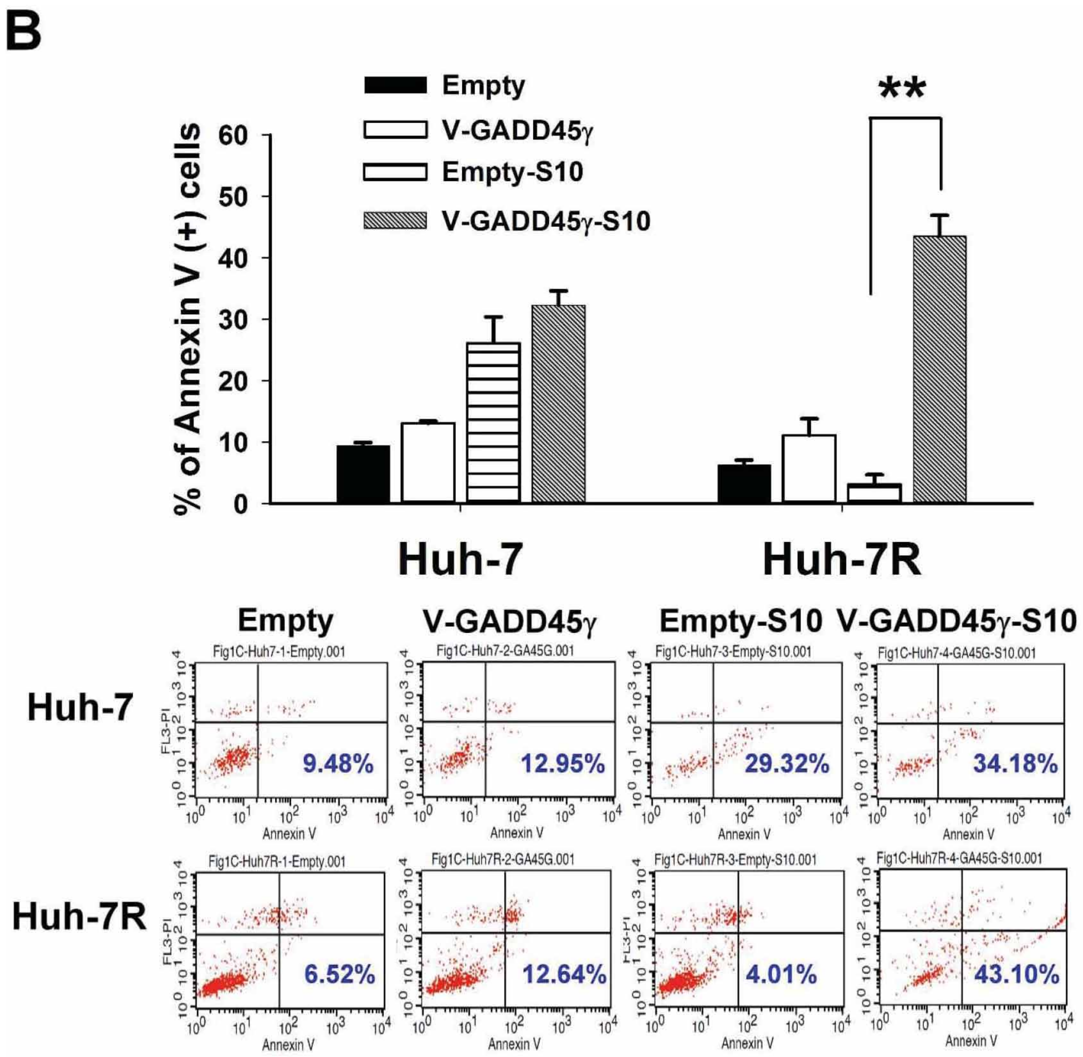

Supplementary Figure S2: (*Continued*) Effects of GADD45γ expression on sorafenib efficacy by Annexin V/PI double staining. B. GADD45γ overexpression could reverse sorafenib resistance. Huh-7 and Huh-7R cells were transfected with GADD45γ or empty vectors and then treated with sorafenib for 48 hours. The proportions of apoptotic cells were measured by Annexin V/PI double staining after 48-hour drug treatment. Proportions of apoptotic cells are indicated by the percentages of Annexin V (+) cells.

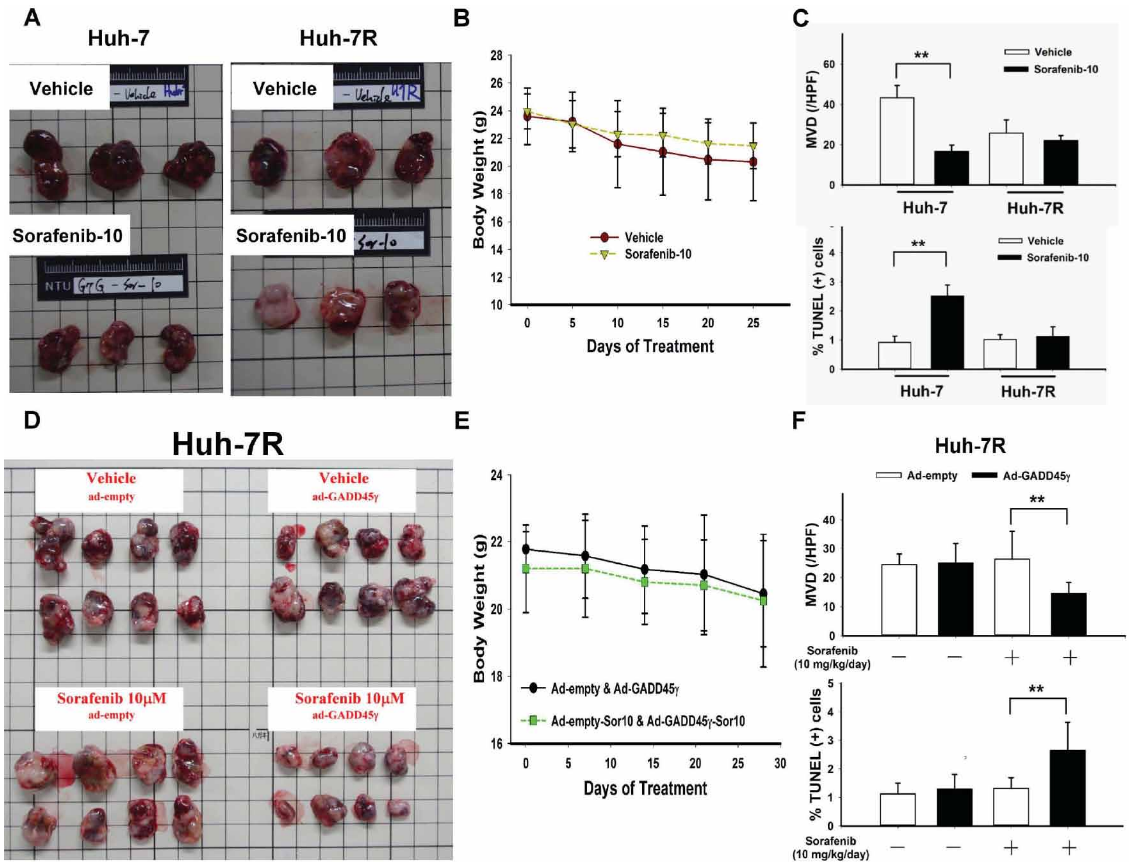

Supplementary Figure S3: GADD45γ induction can reverse sorafenib resistance in xenograft models. A–C. Huh-7 or Huh-7R cells were injected subcutaneously into male BALB/c athymic nude mice. Mice were treated daily by gavage as indicated (V, vehicle; S10, sorafenib 10 mg/kg/day) (n = 5 in each group). (A) Difference in tumor growth after 28 days of sorafenib treatment. (B) Changes in animal body weight (recorded every 5 days). (C) Difference in tumor cell apoptosis and tumor angiogenesis after sorafenib treatment, measured by TUNEL assay and microvessel density (MVD). Quantification of apoptosis and MVD was done by manual counting of TUNEL (+) cells and CD31 (+) microvessels, respectively, under high-power field (HPF, 200×). The numbers were the average of counting 8 HPF in each sample. **, $p < 0.01$, compared with the control (vehicle treated) group. **D–F.** Effects of GADD45γ induction on sorafenib resistance. Huh-7R cells were infected with GADD45γ-expressing or control (10 MOI)-adenoviruses and were implanted subcutaneously into BALB/c athymic (nu+/nu+) mice 24 hours after adenoviral infection. Mice were treated with sorafenib 10 mg/kg/day or vehicle (n = 8 in each group). (D) Difference in tumor growth after 28 days of sorafenib treatment. (E) Changes in animal body weight (recorded every 7 days). (F) Difference in tumor cell apoptosis and tumor angiogenesis after sorafenib treatment in Huh-7R xenografts with or without GADD45γ over-expression, measured by TUNEL assay and microvessel density (MVD). **, $p < 0.01$, compared with the control (Ad-empty) group.

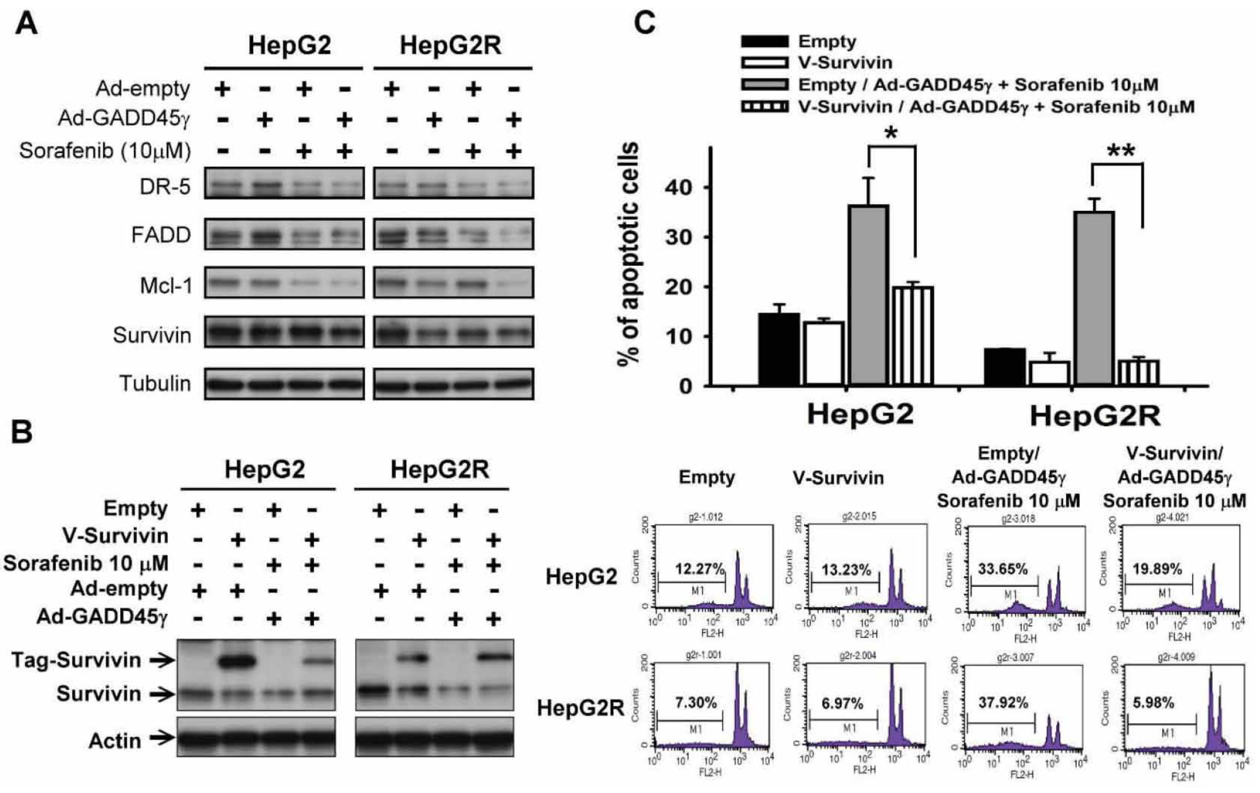

Supplementary Figure S4: GADD45γ induction can reverse sorafenib resistance through survivin inhibition in HepG2 and HepG2R cells. **A.** Effects of GADD45γ induction to reverse sorafenib resistance on apoptosis-related proteins in HepG2 and HepG2R cells. HepG2 and HepG2R cells were infected with GADD45γ-expressing or control adenoviruses and sorafenib (10 μM) for 48 hours. Whole-cell lysates were subjected to Western blotting. **B.** and **C.** Survivin was over-expressed by transfecting pCMV6-Myc-DDK-survivin (V-Survivin) into HepG2 and HepG2R cells. The cells were then treated with sorafenib (10 μM) and GADD45γ-expressing or control adenoviruses. The whole-cell lysates were collected for Western blotting after 48-hour drug treatment. The percentages of apoptotic cells were measured by flow cytometry after 72-hour drug treatment. *, $p < 0.05$, **, $p < 0.01$.

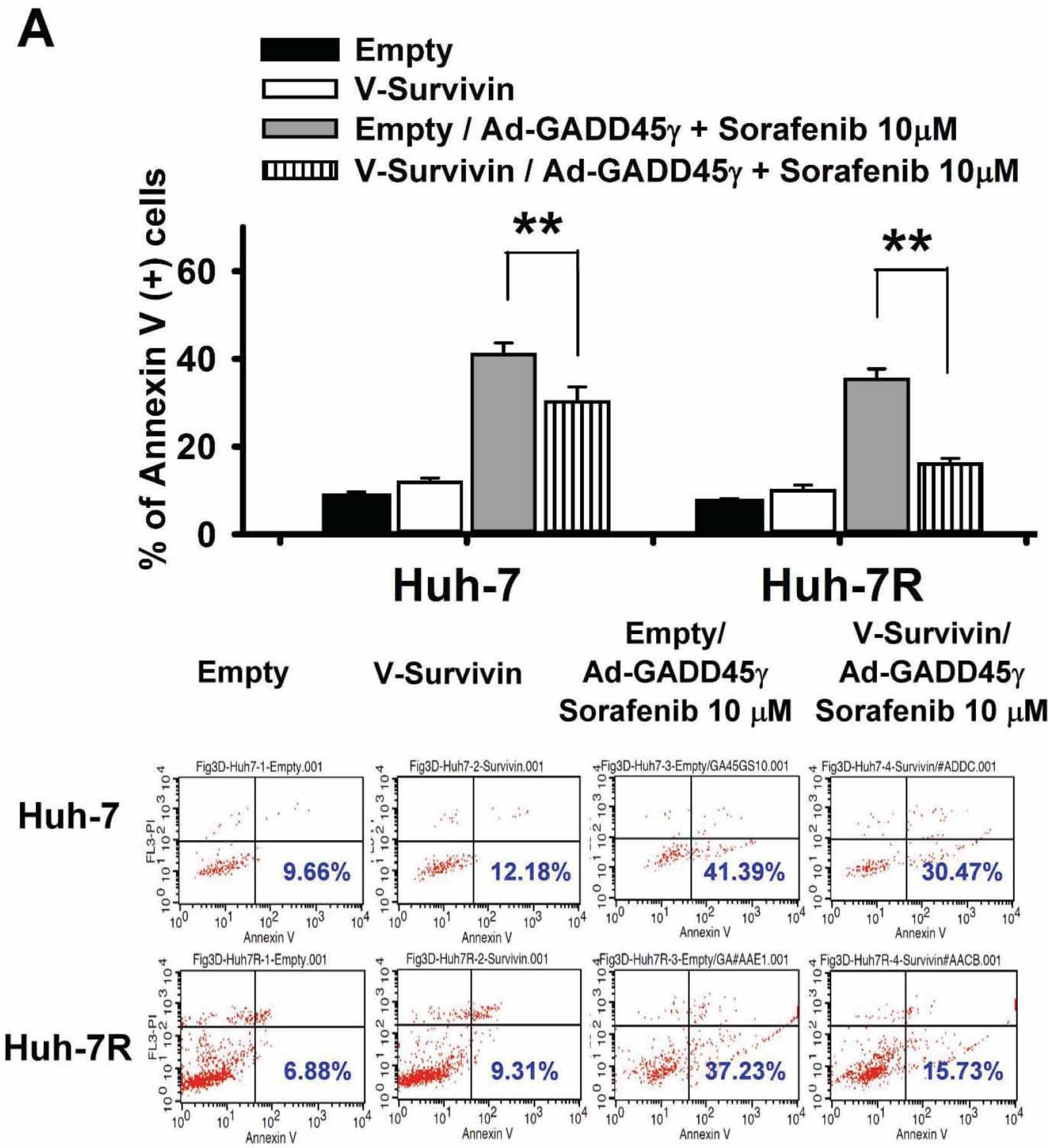

Supplementary Figure S5: Survivin is a mediator of GADD45γ induction to reverse sorafenib resistance by Annexin V/PI double staining. A. Survivin was over-expressed by transfecting pCMV6-Myc-DDK-survivin (V-Survivin) into Huh-7 and Huh-7R cells. The cells were then treated with sorafenib (10 μM) and GADD45γ-expressing or control adenoviruses. (*Continued*)

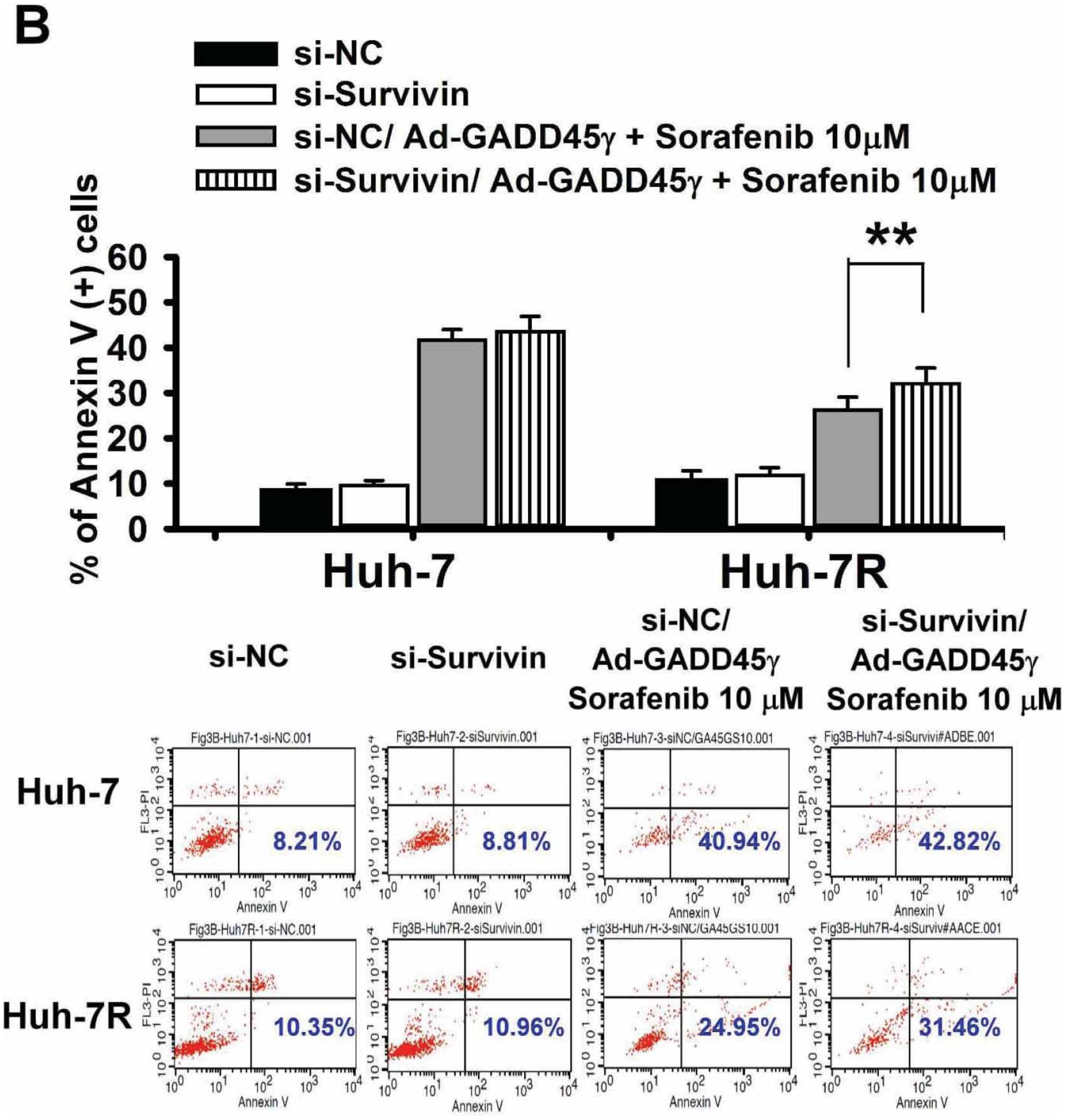

Supplementary Figure S5: (*Continued*) Survivin is a mediator of GADD45γ induction to reverse sorafenib resistance by Annexin V/PI double staining. B. Survivin knockdown enhanced the efficacy of GADD45γ induction combined with sorafenib. Huh-7 and Huh-7R cells were transfected with si-survivin or scrambled siRNA for 12 hours. The cells were then treated with sorafenib (10 μM) and GADD45γ-expressing or control adenoviruses. The proportions of apoptotic cells were measured by Annexin V/PI double staining after 48-hour drug treatment. Proportions of apoptotic cells are indicated by the percentages of Annexin V (+) cells.

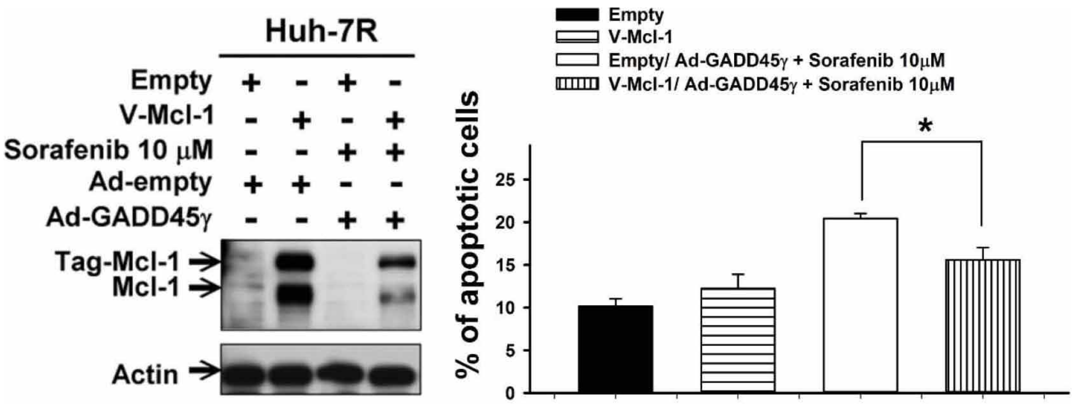

Supplementary Figure S6: GADD45γ induction can partially reverse sorafenib resistance through Mcl-1 inhibition. Survivin was over-expressed by transfecting pCMV6-Myc-DDK-Mcl-1 (V-Mcl-1) into Huh-7R cells. The cells were then treated with sorafenib (10 μM) and GADD45γ-expressing or control adenoviruses. The whole-cell lysates were collected for Western blotting after 48-hour drug treatment. The percentages of apoptotic cells were measured by flow cytometry after 72-hour drug treatment. *, $p < 0.05$.

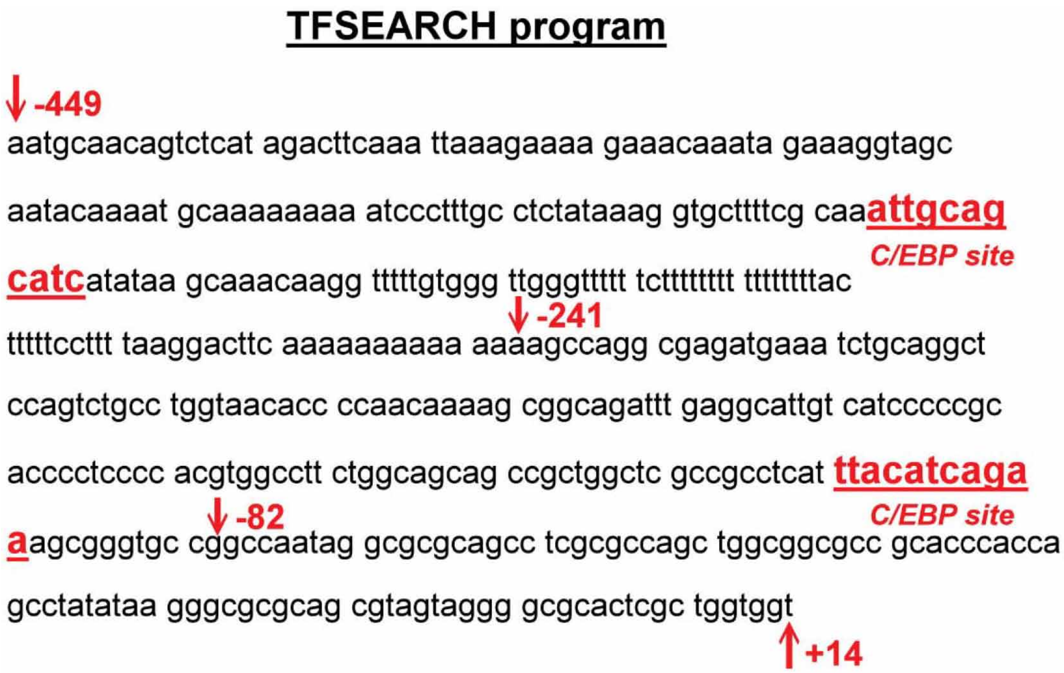

Supplementary Figure S7: Sequence of the -449/-82 region of the human GADD45γ promoter. The CEBP (-340/-330) and (-103/-93) binding sites, identified by the TFSEARCH program, are underlined.

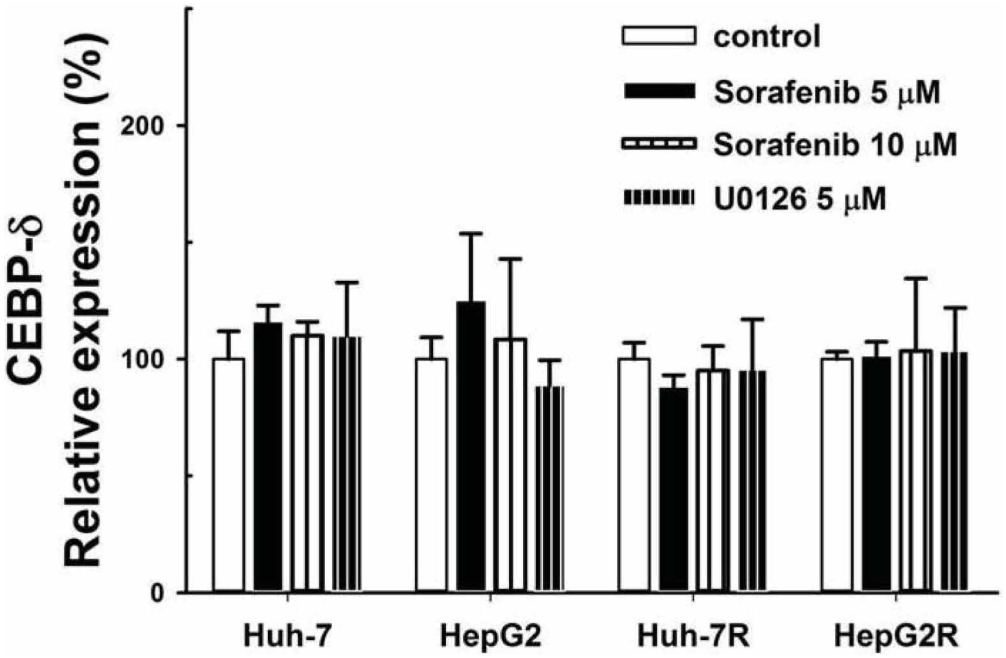

Supplementary Figure S8: C/EBPδ mRNA expression was not affected by sorafenib and U0126 (MEK inhibitor) in HCC cell lines. C/EBPδ mRNA was examined after sorafenib or U0126 treatment by real-time qRT-PCR in HCC cell lines. The relative mRNA amount of the C/EBPδ / HPRT (endogenous control) genes was calculated using the ΔCt (threshold cycle) method: relative expression = 2−ΔCt, where ΔCt = Ct (target gene) − Ct (control gene).

Supplementary Table S1: Primers for quantitative real time PCR, reporter assay and ChIP assay

qRT-PCR

HPRT		Sense	5′-TGACACTGGCAAAACAATGCA-3′
		Anti-sense	5′-GGTCCTTTTCACCAGCAAGCT-3′
siRNA			
GADD45γ-1		Sense	5’-CGAGAACGACAUCGACAUAtt-3’
		Anti-sense	5’-UAUGUCGAUGUCGUUCUCGca-3’
GADD45γ-2		Sense	5’-CUGUUGAAGCUUUGAAUUUtt-3’
		Anti-sense	5’-AAAUUCAAAGCUUCAACAGca-3’
Mcl-1		Sense	5’-CCAGUAUACUUCUUAGAAAtt-3’
		Anti-sense	5’-UUUCUAAGAAGUAUACUGGga-3’
GADD45γ promoter construct			Deletion fragments of GADD45γ promoter (The *EcoRV* and *HindIII* sites were underlined)
		Sense	5’- AACA<u>GATATC</u>CGGCGTTGTTTACTTTCC -3’ (starting at –1711)
			5’-TTTT<u>GATATC</u>CTTACTCGGGTTCTATTT -3’ (–1281)
			5’-GGCA<u>GATATC</u>CGACCCCCACGCGCGGCG -3’ (–853),
			5’-AATG<u>GATATC</u>TCTCATAGACTTCAAATT -3’ (–451),
			5’-AAGC<u>GATATC</u>AGATGAAATCTGCAAGGCT-3’ (–241),
			5’-GCC<u>GATATC</u>CGCGCAGCCTCGCGCCAG-3’ (–82),
		Anti-sense	5’-ACCA<u>AAGCTT</u>AGTGCGCCCCTACTACGC-3’
		Mutated at C/EBP (C/EBPm)	Mutated at C/EBP (C/EBPm): ↓-449<br>aatg<u>GATATC</u>tctcat agacttcaaa ttaaagaaaa gaaacaaata gaaaggtagc<br>*EcoR V*<br>aatacaaaat gcaaaaaaaa atccctttgc ctctataaag gtgcttttcg caa<u>CCCCTTT</u><br>*C/EBPm site*<br><u>TGGA</u>atataa gcaaacaagg tttttgtggg ttgggttttt tctttttttt tttttttac<br>↓-241<br>tttttccttt taaggacttc aaaaaaaaaa aaaagccagg cgagatgaaa tctgcaggct<br>ccagtctgcc tggtaacacc ccaacaaaag cggcagattt gaggcattgt catcccccgc<br>acccctcccc acgtggcctt ctggcagcag ccgctggctc gccgcctcat <u>CCCTTTTCCC</u><br>↓-82 *C/EBPm site*<br><u>C</u>agcgggtgc cggccaatag gcgcgcagcc tcgcgccagc tggcggcgcc gcacccacca<br>gcctatataa gggcgcgcag cgtagtaggg gcgcact<u>TTCGAA</u>gtggt<br>*Hind III* ↑+14<br>ChIP assay — Sense: 5'- ACAGTCTCATAGACTTCAAATT -3' (-443)<br>Anti-sense: 5'-CGAGGCTGCGCGCCTATTG -3' (-61)
ChIP assay		Sense	5’- ACAGTCTCATAGACTTCAAATT -3’ (–443)
		Anti-sense	5’-CGAGGCTGCGCGCCTATTG -3’ (–61)

Supplementary Table S2: Uni-variate analysis of overall survival and recurrence-free survival

	Overall survival		Recurrence-free survival	
	HR	*P* value	HR	*P* value
**Age < 65 y vs. ≧ 65 y**	**0.33**	**0.007**	**0.47**	**0.017**
**MVI (-) vs. (+)**	**0.31**	**0.005**	**0.30**	**< 0.0001**
**Sex male vs. female**	1.20	0.390	1.17	0.644
**AFP (ng/mL)**	1.00	0.559	1.00	0.359
**Stage IIIA vs. I/II**	2.16	0.084	**2.76**	**0.004**
**Cirrhosis (-) vs. (+)**	1.00	0.999	0.96	0.912
**Tumor grade 2 vs. 0-1**	1.31	0.566	1.04	0.901
**HBV vs. HCV**	0.96	0.849	0.94	0.838
**GADD45γ RNA ≧ median vs. < median**	**0.48**	**0.072**	0.82	0.487

Supplementary Table S3: Correlation between GADD45γ expression and survival of HCC patients from the cBio Portal for Cancer Genomics database (http://www.cbioportal.org)

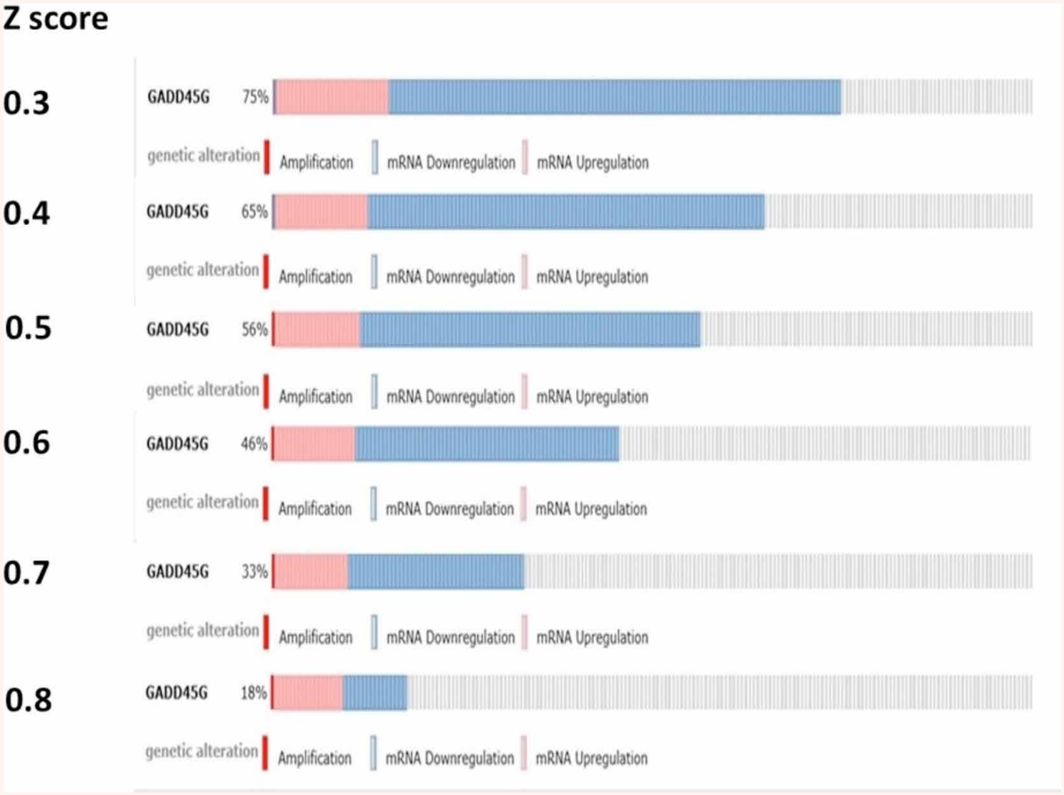

In Z scores 0.9 to 2.0, all alterations in GADD45γ expression were amplification/ over-expression. A consistent trend of superior survival was seen in patients with GADD45γ amplification/ over-expression, as listed below.

(*Continued*)

		Overall survival (months)		Disease-free survival (months)	
**Z-score threshold**	**Percentage of patients with GADD45γ amplification/ over-expression**	**Median (with vs. without amplification/ over-expression)**	***P* value**	**Median (with vs. without amplification/ over-expression)**	***P* value**
0.9	8.9	70.0 vs.23.3	0.012	90.1 vs. 18.4	0.011
1.0	7.9	70.0 vs.23.3	0.012	90.1 vs. 18.4	0.011
1.1	6.3	70.0 vs.23.3	0.030	90.1 vs. 18.4	0.012
1.2	5.8	70.0 vs.25.3	0.030	90.1 vs. 18.4	0.012
1.3 – 1.6	5.3	70.0 vs.25.3	0.030	NA vs. 18.4	0.021
1.7	4.2	70.0 vs.25.3	0.068	NA vs. 19.2	0.056
1.8 – 2.0	3.2	70.0 vs.25.3	0.086	NA vs. 19.2	0.070
Representative survival curves were shown below.					

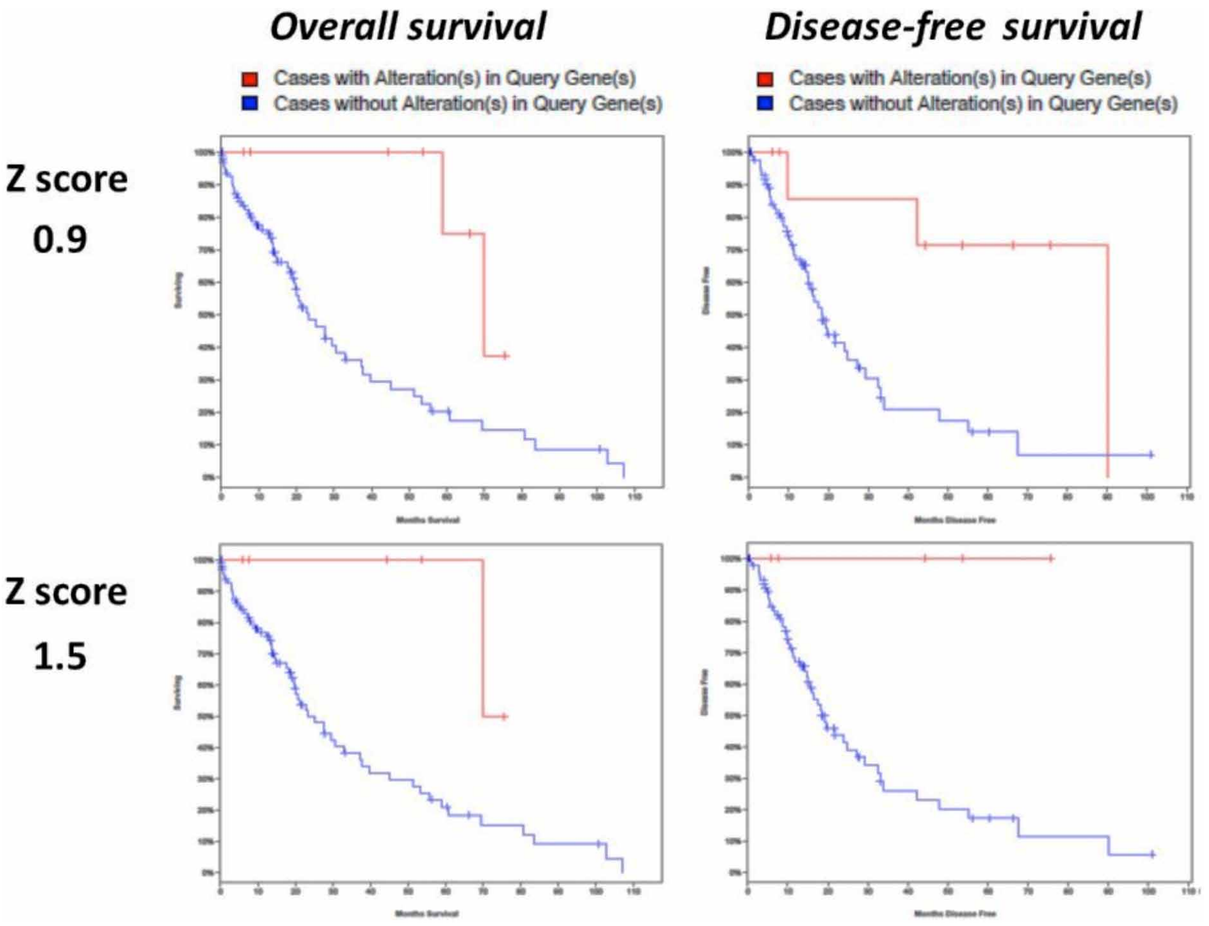

The case set consisted of 190 HCC samples that have mRNA, CAN, and sequencing data (accessed on 25 January 2015).
A sensitivity test using Z score from 0.3 to 2.0 was done. In Z scores 0.3 to 0.8, both up- and down-regulation were classified as 'alterations' in GADD45γ expression, as shown below, and it was thus difficult to compare survival in patients with or without alterations in GADD45γ expression according to the instructions from the cBio Portal website.
